# Early manifestations of genetic liability for ADHD, autism and schizophrenia at ages 18 and 24 months

**DOI:** 10.1002/jcv2.12093

**Published:** 2022-07-21

**Authors:** Lucy Riglin, Esther Tobarra‐Sanchez, Evie Stergiakouli, Alexandra Havdahl, Kate Tilling, Michael O’Donovan, Joel Nigg, Kate Langley, Anita Thapar

**Affiliations:** ^1^ Division of Psychological Medicine and Clinical Neurosciences and MRC Centre for Neuropsychiatric Genetics and Genomics Cardiff University Cardiff UK; ^2^ Wolfson Centre for Young People's Mental Health Cardiff UK; ^3^ MRC Integrative Epidemiology Unit University of Bristol Bristol UK; ^4^ Population Health Sciences Bristol Medical School University of Bristol Bristol UK; ^5^ Nic Waals Institute Lovisenberg Diaconal Hospital Oslo Norway; ^6^ Department of Mental Disorders Norwegian Institute of Public Health Oslo Norway; ^7^ PROMENTA, Department of Psychology University of Oslo Oslo Norway; ^8^ Deptartment of Psychiatry Oregon Health & Science University Portland Oregon USA; ^9^ School of Psychology Cardiff University Cardiff UK

**Keywords:** ADHD, ALSPAC, antecedents, autism, development, schizophrenia, temperament

## Abstract

**Background:**

ADHD and autism are neurodevelopmental conditions, for which non‐specific precursors or early signs include difficulties with language and motor skills, and differences in temperament in the first and second year of life. These early features have also been linked to later diagnosis of schizophrenia which is widely considered to have neurodevelopmental origins. Given that ADHD, autism and schizophrenia are all highly heritable, we tested the hypothesis that in the general population, measures of toddler language development, motor development and temperament are associated with genetic liability to ADHD, autism and/or schizophrenia.

**Methods:**

Data were analysed from the Avon Longitudinal Study of Parents and Children (ALSPAC) which included motor development scores at age 18 months and language development and temperament scores at age 24 months (*N* = 7498). Genetic liability was indexed by polygenic risk scores (PGS) for ADHD, autism and schizophrenia.

**Results:**

ADHD PGS were associated with specific temperament scales (higher activity *β* = 0.07, 95% CI = 0.04, 0.09 and lower withdrawal *β* = −0.05, 95% CI = −0.07, −0.02) as well as better gross motor scores (*β* = 0.04, 95% CI = 0.01, 0.06). Schizophrenia PGS were associated with one specific temperament scale (negative mood *β* = 0.04, 95% CI = 0.02, 0.07). We did not find strong evidence of association of autism PGS with any of the toddler measures; there was also not strong evidence of association with motor or language delays for any of the PGS.

**Conclusions:**

This study suggests that some specific aspects of early temperament and gross motor differences in the general population could represent part of the early manifestation of genetic liability to neurodevelopmental conditions.

## Graphical abstract

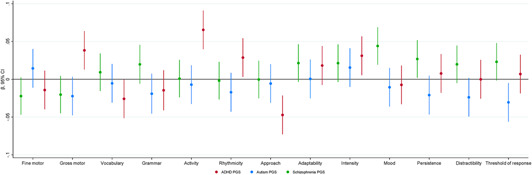

We investigated associations for genetic liability to ADHD, autism and schizophrenia with motor, language and temperament scores at 18–24 months. We found ADHD genetic liability to be associated with better gross motor scores, higher activity and lower withdrawal and schizophrenia genetic liability to be associated with increased negative mood; we did not find strong evidence of association for autism genetic liability. Results suggest that specific aspects of toddler temperament and gross motor differences in the general population could represent part of the early manifestation of genetic liability to neurodevelopmental conditions.

**Key points**

- Precursors or early signs of ADHD, autism and schizophrenia include difficulties with language and motor skills, and differences in temperament in the first 2 years of life
- We investigated associations for genetic liability to ADHD, autism and schizophrenia with motor, language and temperament scores at 18–24 months
- We found ADHD genetic liability to be associated with better gross motor scores, higher activity and lower withdrawal and schizophrenia genetic liability to be associated with increased negative mood; we did not find strong evidence of association for autism genetic liability
- Specific aspects of toddler temperament and gross motor differences in the general population could represent part of the early manifestation of genetic liability to neurodevelopmental conditions

## INTRODUCTION

As currently conceptualised in DSM‐5 (American Psychiatric Association, 2013), childhood‐onset neurodevelopmental conditions, such as Attention Deficit Hyperactivity Disorder (ADHD) and Autism Spectrum Disorder (hereafter, autism), arise in early development (Thapar et al., 2017). Autism typically manifests in the first 3 years of life although it may not be clinically recognised until later (Thapar & Rutter, 2015). ADHD tends to present later; although its core clinical features also typically manifest in the preschool years (Nigg, 2006). Several precursors or early signs of these highly heritable conditions have been observed in prospective longitudinal studies. Delays in motor development including fine motor skills have been observed in those who go on to be diagnosed with autism (Bolton et al., 2012; Bussu et al., 2018; Jaspers et al., 2013; Johnson et al., 2015), but this is less commonly reported in ADHD (Johnson et al., 2015), where findings appear more heterogeneous such that advanced as well as delayed motor development have been found (Athanasiadou et al., 2020; Havmoeller et al., 2019; Jaspers et al., 2013; Lemcke et al., 2016). Early language development delays including poor vocabulary and grammar have been observed in people with autism (Bolton et al., 2012) and ADHD (Arnett et al., 2013; Lemcke et al., 2016), although some work suggests language (as well as psychomotor) delays are more common in autism than in ADHD (Bolton et al., 2012; Jaspers et al., 2013). Differences in early temperament have also been observed in people with ADHD and autism: in toddlerhood these have included lower approach and adaptability, and greater negative affect and perceptual sensitivity in autism (Clifford et al., 2013; Del Rosario et al., 2014; Johnson et al., 2015; Zwaigenbaum et al., 2005), and higher levels of activity and approach (being less cautious) and lower levels of effortful control in ADHD (Del Rosario et al., 2014; Johnson et al., 2015; Kostyrka‐Allchorne et al., 2020; Lemcke et al., 2016; Nigg et al., 2020).

Although schizophrenia is not listed in the neurodevelopmental section of the DSM‐5 and has median age of onset in late adolescence, it is also highly heritable and is widely considered to have neurodevelopmental origins (Pino et al., 2014; Rapoport et al., 2005). In common with autism and ADHD, some individuals with schizophrenia have a history of delayed motor and language development (Fryers & Brugha, 2013; Isohanni et al., 2004; Rutter et al., 2006). Although there is a dearth of research investigating associations between toddler temperament and schizophrenia, research also suggests association with prior social and emotional difficulties (Pine & Fox, 2015; Rutter et al., 2006).

Such deviations from typical development could represent the earliest manifestations of these neurodevelopmental conditions. They could also reflect pleiotropic effects of neurodevelopmental risk alleles on multiple areas of functioning at different developmental periods (Wray et al., 2014). These would suggest that neurodevelopmental risk alleles would be associated with development in the general population and either explanation would suggest potential benefit of heightened clinical vigilance and monitoring for the emergence of neurodevelopmental conditions in toddlers, children and adolescents who display early developmental difficulties.

Genetic liability to neurodevelopmental conditions may be mediated through these early non‐specific features, but this possibility has been little studied. Work in the Dutch Generation R population‐based sample found evidence of association with delays in different aspects of neuromotor development in early infancy (9–20 weeks) for ADHD, autism and schizophrenia common gene variants as indexed by polygenic scores (PGS) (Serdarevic et al., 2018; Serdarevic et al., 2020). Work in the Norwegian population‐based MoBa cohort examining associations between ADHD, autism and schizophrenia PGS with motor and language difficulties at multiple ages (spanning 6 months to 8 years) found that before 2 years of age, robust evidence for association was limited to autism PGS and language difficulties (Askeland et al., 2021); further work examining age at first walking found ADHD PGS to be associated with earlier age at walking and autism PGS to be associated with later age at walking (in females) but with no robust associations with age at first words or phrases (Hannigan et al., 2021). To our knowledge, no studies have yet examined the relationships between genetic liability to ADHD, autism and schizophrenia and temperament in the toddler years, despite ample theory and prior prospective and family and twin studies suggesting links between early temperament and later neurodevelopmental and psychiatric disorders (Nigg et al., 2020).

We set out to test the hypothesis that PGS for ADHD, autism and schizophrenia in the general population would be associated with motor and language developmental difficulties and temperamental differences in toddlerhood (age 18–24 months). Based upon the literature summarised above, we hypothesised: (a) ADHD PGS would be associated with higher levels of activity and approach and lower effortful control, (b) autism and schizophrenia PGS would be associated with poorer motor and language development, higher withdrawal (lower approach) and greater negative affect, and (c) autism PGS would be associated with lower adaptability (behavioural flexibility in a changing context).

## METHODS

### Sample

We analysed data from the Avon Longitudinal Study of Parents and Children (ALSPAC), a well‐established prospective, longitudinal birth cohort study. Pregnant women resident in Avon, UK with expected dates of delivery 1st April 1991 to 31st December 1992 were invited to take part in the study. Of these initial pregnancies 13,988 children were alive at 1 year of age (Boyd et al., 2013; Fraser et al., 2013; Northstone et al., 2019). Our sample included European individuals with genetic data (see below, one child per family) who were alive at 1 year: *N* = 7498; for our primary analyses sample size varied from *N* = 5880–6328 depending on availability of the outcome variable (see below and Table S1). Full details of this study are provided in the Supporting Information.

### Genetic data

Polygenic scores (PGS) were generated as the weighted mean number of disorder risk alleles in approximate linkage equilibrium, derived from imputed autosomal SNPs using PRSice (Euesden et al., 2015). Scores were standardized using Z‐score transformation. Risk alleles were defined as those associated with case‐status in recent large consortia analyses of ADHD (19,099 cases and 34,194 controls) (Demontis et al., 2019), autism (18,381 cases and 27,969 controls) (Grove et al., 2019), and schizophrenia (40,675 cases and 64,643 controls) (Pardinas et al., 2018). A principal component approach was used to examine associations with PGS (the PGS‐PCA approach) whereby (a) PGS were calculated using seven different *p*‐value thresholds, (b) the first principal component was extracted based on the correlation matrix for the seven different PGS, and (c) the first principal component was used to examine associations between PGS and motor, language and temperament outcomes (see below). This method avoids multiple testing associated with using multiple PGS derived using different *p*‐value thresholds aims to extract the maximum variation without overfitting (Coombes et al., 2020). Genotyping details as well as full methods for generating the PGS are presented in the Supporting Information.

### Motor development

Fine and gross motor skills were assessed by parent‐report at child age 18 months using an adapted version of the Denver Developmental Screening Test (Frankenburg & Dodds, 1967; Iles‐Caven et al., 2016). Parents were asked to assess whether the study child had reached 16 fine motor and 12 gross motor milestones on a three‐point scale (0 = not yet started, 1 = only done 1–2 times, 2 = yes, can do well). Analysed motor scores were age adjusted z‐scores.

### Language development

Vocabulary and grammar were assessed by parent‐report at child age 2 years using an adapted version of the MacArthur Communicative Development Inventory (Feldman et al., 2000; Law et al., 2019). To assess vocabulary, parents were asked whether the study child understands/says 123 words spanning 19 semantic categories on a three‐point scale (0 = neither, 1 = understands, 2 = says), summed to give a vocabulary score (possible range 0–246). To assess grammar, parents were asked whether the study child had begun to use 4 plural words on a three‐point scale (0 = not yet, 1 = sometimes, 2 = often) and whether the child understands/says 5 additional plural words and 20 past tense words on a three‐point scale (0 = neither, 1 = understands, 2 = says), summed to give a grammar score.

### Temperament

Temperament was assessed by parent‐report at child age 2 years using the Carey Temperament Scale (Carey & McDevitt, 1978) that included nine subscales (Chess & Thomas, Hepburn & Stone, 2006): activity (motor component present in a child's functioning: 9‐items), rhythmicity (irregularity; unpredictability in daily functions: 11‐items), approach (withdrawal; initial response to a novelty: 11‐items), adaptability (low behavioural flexibility in changing context: 7‐items), intensity (energy level of an emotional response: 9‐items), mood (tone of overall affect, negative: 12‐items), persistence (low continuation of activity in face of obstacles: 9‐items), distractibility (effectiveness of extraneous stimuli in altering the direction of ongoing behaviour: 10‐items) and threshold of responsiveness (intensity level of stimulation necessary to evoke discernible response: 8‐items), assessed on a five‐point scale (0 = almost never, 1 = rarely, 2 = sometimes, 3 = often, 4 = almost always) summed to give total subscale scores. Scale reliabilities and the interpretations of low and high subscale scores are shown in Table 1.

**TABLE 1:** Interpretation of low and high temperament scores

	Lower score	Higher score	Items	Reliability (α)
Activity	Lower activity	Higher activity	9	0.75
Rhythmicity (irregularity)	Lower irregularity	Higher irregularity	11	0.73
Approach (withdrawal)	Lower withdrawal	Higher withdrawal	11	0.81
Adaptability (flexibility)	Lower flexibility	Higher flexibility	7	0.69
Intensity	Lower intensity	Higher intensity	9	0.76
Mood	Lower negative mood	Higher negative mood	12	0.76
Persistence	Higher persistence	Lower persistence	9	0.76
Distractibility	Lower distractibility	Higher distractibility	10	0.82
Threshold of response	Lower threshold of response	Higher threshold of response	8	0.65

*Note*: Correlations between subscales are shown in Table S3. Reliability assessed by Cronbach's alpha.

### Analyses

Multivariable linear regressions were conducted to investigate associations between the three PGS (simultaneously). Each of the continuous outcomes were investigated separately; to correct for multiple testing of 13 outcomes, we used the Bonferroni method to correct the *p*‐value threshold of 0.05 assuming 11 independent tests (the number of principle components explaining 95% of the variance in the outcomes (Bell et al., 2018): see Table S2): multiple testing corrected *p*‐value threshold was therefore set at 0.05/11 = 0.005. Additional covariates were not included in primary analyses. Secondary analyses were conducted stratified by sex. Sensitivity analyses conducted (i) univariate analyses (i.e. not covarying for each of the PGS), (ii) using inverse probability weighting (Seaman & White, 2013) (IPW) to assess the impact of missing genetic data (see Supporting Information), and (iii) including population stratification covariates (see Supporting Information).

## RESULTS

**FIGURE 1:**
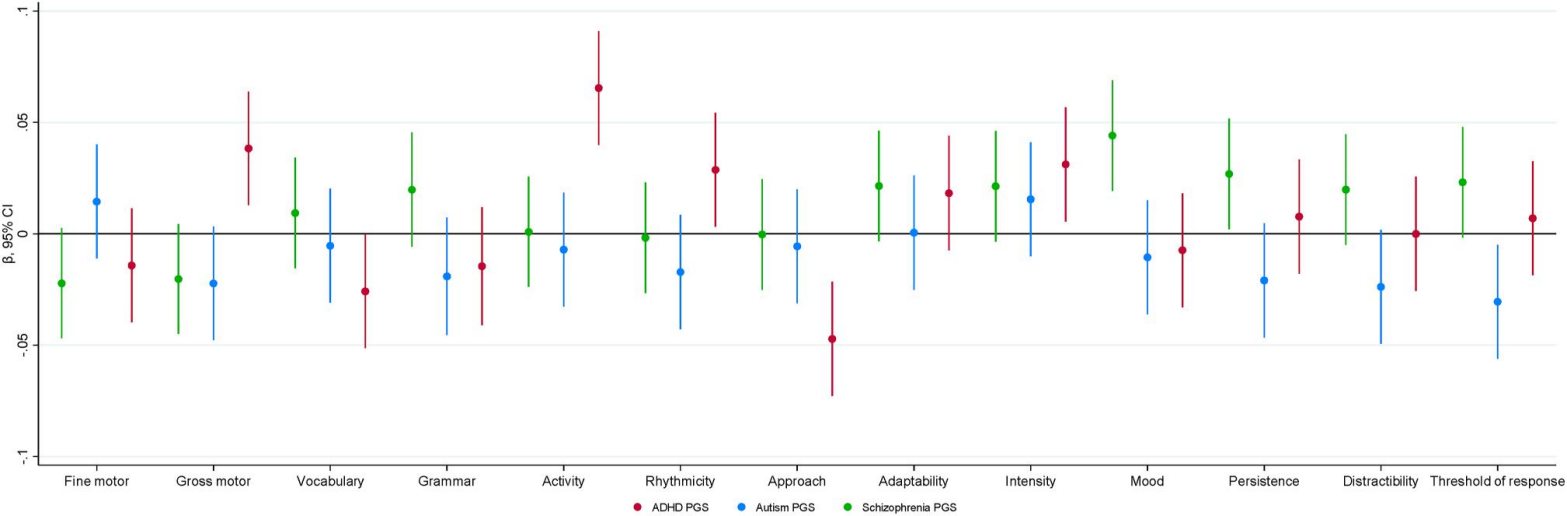
Multivariable associations between polygenic scores (PGS) and toddler developmental outcomes

ADHD PGS correlated with autism PGS and schizophrenia PGS at *r* = 0.24 (95% CI = 0.22–0.27) and *r* = 0.06 (95% CI = 0.04–0.08) respectively; autism PGS and schizophrenia PGS were correlated at *r* = 0.04 (95% CI = 0.02–0.07). Correlations between the 13 toddler outcomes are shown in Table S3 and ranged from 0 to ±0.81. Multivariable associations between ADHD, autism and schizophrenia PGS and the early motor, language, and temperamental measures are shown in Figure 1 and Table S1.

ADHD PGS were associated with higher gross motor (*β* = 0.04, 95% CI = 0.01, 0.06), higher activity (*β* = 0.07, 95% CI = 0.04, 0.09) and lower withdrawal scores (*β* = −0.05, 95% CI = −0.07, −0.02). Schizophrenia PGS were associated with higher (negative) mood scores (*β* = 0.04, 95% CI = 0.02, 0.07). We did not find strong evidence of associations for autism PGS.

### Sex differences

**FIGURE 2:**
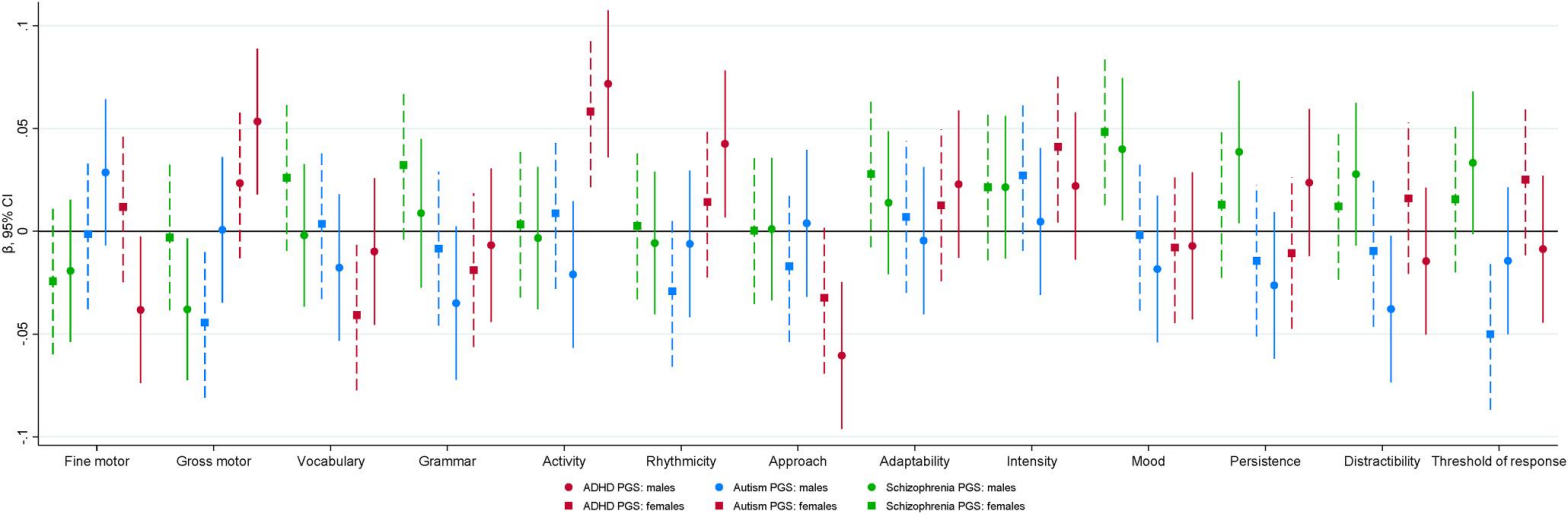
Multivariable associations between polygenic scores (PGS) and toddler developmental outcomes stratified by sex

Sex‐stratified analyses are shown in Figure 2 and Table S4: associations for ADHD PGS with higher gross motor and lower withdrawal scores were somewhat more apparent in males (*β* = 0.05, 95% CI = 0.02, 0.09 and *β* = −0.06, 95% CI = −0.10, −0.02 respectively) than females (*β* = 0.02, 95% CI = −0.01, 0.06 and *β* = −0.03, 95% CI = −0.07, 0.00 respectively) and associations between schizophrenia PGS and negative mood were somewhat more apparent in females (*β* = 0.05, 95% CI = 0.01, 0.08) than males (*β* = 0.04 95% CI = 0.07, 0.07); however there was not strong evidence that effect sizes differed by sex (all interaction p‐values ≥ 0.05: see Table S5).

### Sensitivity analyses

A similar pattern of results was observed for univariable analyses (i.e. when PGS were not entered simultaneously: Table S6), for analyses conducted using IPW (Table S7) and when including population stratification covariates (Table S8) suggesting results were not substantially impacted by covariance between PGS, or by bias arising from missing PGS data or by population stratification.

Sensitivity analyses were conducting excluding individuals who developed ADHD (detailed in the Supporting Information): these results suggested associations observed in our primary analyses were now driven by ADHD cases (see Table S9).

## DISCUSSION

This population‐based study set out to examine associations between genetic liability to neurodevelopmental conditions (ADHD, autism and schizophrenia), as indexed by polygenic scores, with toddler motor, language and temperamental development that are potential early precursors or indicators of later neurodevelopmental disorder. As hypothesised, we found evidence of association for ADHD PGS with maternal rated activity levels (as well as with increased (better) gross motor scores), although not with indicators of effortful control. We did not find strong evidence of association with motor or language development or any temperament domains for autism PGS. Schizophrenia PGS was associated with negative affect.

Our findings that ADHD PGS are associated with higher levels of activity and approach in toddlerhood are consistent with previous work on ADHD itself (Johnson et al., 2015; Nigg et al., 2020). These are related to the temperament superfactor of surgency/extraversion, and thus also correlated with impulsivity in childhood. These associations are also consistent with previous work showing ADHD genetic liability to be associated with pre‐school hyperactive/impulsive and inattentive behaviour in the general population (Groen‐Blokhuis et al., 2014), and the high genetic correlation (approximately rG = 1) between diagnoses ADHD and ADHD traits in the population (Demontis et al., 2019) although we focus on an earlier age. We did not find strong evidence of association with indicators of effortful control (persistence and distractibility) at this early age even though ADHD PGS are associated with later ADHD traits in this population cohort (Martin et al., 2014).

In addition to associations with measures of temperament, we found ADHD PGS to be associated with higher gross motor scores, consistent with findings in MoBa of association between ADHD PGS and earlier age at walking (Hannigan et al., 2021) and not with findings in Generation R of association between ADHD PGS and motor delay (Serdarevic et al., 2020). Reported associations between ADHD itself and motor delay are mixed, with evidence suggesting that some individuals with ADHD show delayed motor development whilst others show advanced motor development (Athanasiadou et al., 2020; Havmoeller et al., 2019; Jaspers et al., 2013; Lemcke et al., 2016). It may be that different subgroups show diverse patterns of association with motor delay or it may be that our measure (see Table S10) captures motor activity (e.g. the frequency of activity) as well as motor delay, which are more easily differentiated by formal in‐person assessments. Further investigation in different populations, comparing different types of assessments is warranted.

We hypothesised that autism and schizophrenia PGS would be associated with poorer motor and language development (Bolton et al., 2012; Bussu et al., 2018; FryersT & Brugha, 2013; Isohanni et al., 2004; Jaspers et al., 2013; Johnson et al., 2015; Rutter et al., 2006) but did not find strong evidence to support that prediction. Previous work in a smaller Dutch sample found infant motor development assessed by home visit examinations earlier in infancy (age 9–20 weeks) to be associated with autism PGS and schizophrenia PGS (Serdarevic et al., 2018; Serdarevic et al., 2020). Work in the larger Norwegian MoBa cohort found strong evidence of association between autism PGS and motor difficulties only at age 3 years (with consistent but weaker evidence of association at 6 and 18 months, and 5 years), as well as with language difficulties at 18 months (with consistent but weaker evidence of association at 3, 5 and 8 years) (Hannigan et al., 2021). They found differences in associations depending on the aspect of motor and language development that was measured, with strong evidence of association between autism PGS and later age at walking in females, but no strong evidence of association with motor delay at age 18 months (Hannigan et al., 2021). Age at walking could be a more sensitive measure of early subtle differences in motor development than measures of motor delays or difficulties. It also may be that the genetic contribution to atypical motor and language development characteristics of many neurodevelopmental conditions is different to the genetic factors that contribute to variation in language development in the general population. Another explanation may be that specific language problems such as pragmatic use of language are not encompassed within the questionnaires utilised here or that these become more prominent later during school years.

Based on previous work on temperament we also hypothesised that autism PGS would be associated with higher withdrawal, greater negative affect and lower adaptability (Clifford et al., 2013; Del Rosario et al., 2014; Johnson et al., 2015; Zwaigenbaum et al., 2005), but we did not find strong evidence of association with any of these temperament domains. This could be because temperament profiles associated with autism liability become somewhat more distinct across toddlerhood (Del Rosario et al., 2014), because the observed associations between facets of temperament and autism are not driven by shared genetic risk or because the shared genetic risk is not captured by autism PGS, or because any associations are small. Finally, based on associations between schizophrenia and premorbid social and emotional difficulties (Pine & Fox, 2015; Rutter et al., 2006) we hypothesised schizophrenia PGS would be associated with higher withdrawal and greater negative affect: we only found strong evidence of association with negative affect.

Overall, we found more support for our hypotheses relating to ADHD than autism or schizophrenia PGS. The findings suggest that ADHD PGS are associated with ADHD‐like behaviours very early in life, years before a diagnosis of ADHD would typically be considered. Interestingly our findings on early development and temperament contrast with findings on this same cohort when they are older: previous studies have found that ADHD, autism and schizophrenia PGS are associated with measures of psychopathology and behaviour during childhood, adolescence and early adult life (Jones et al., 2016; Riglin et al., 2017; Riglin et al., 2020; St Pourcain et al., 2018).

Strengths of this study include a large population‐based cohort and prospective design. However, weaknesses include the restriction to parent‐ratings of development and attrition. Future studies could benefit from more rigorous measures of development with clinical measures of atypical development rather than developmental screeners. In this regard, high‐risk studies may be of considerable value as these will be enriched for toddlers and children with atypical developmental profiles at risk for neurodevelopmental conditions. Polygenic scores are weak predictors and our measures of genetic liability (polygenic scores) also only capture a small proportion of variance. Whilst larger GWAS will lead to more powerful PGS, most agree that on their own they are unlikely to be clinically relevant predictors but could in future be combined with clinical variables (Murray et al., 2021). Our failure to observe associations with ASD PRS when associations with ADHD PRS were detected could reflect differences in power: the ASD GWAS discovery sample is small and common variants in the latest ASD GWAS (Grove et al., 2019) generate lower SNP‐heritability estimates than for ADHD. SNP‐heritability places an upper bound on PGS effect sizes; while SNP heritability was not available for the phenotypes studied here, low SNP heritability could have contributed to some non‐detection findings. We note that our polygenic scores were generated using PRSice (Euesden et al., 2015) and other methods such as LDpred2 (Privé et al., 2020) may have given slightly different results. Our polygenic score approach also cannot differentiate whether motor development and temperament lie on the causal pathway between genetic liability to neurodevelopmental conditions and later neurodevelopmental conditions, if these associations arise independently as the result of (horizontal) pleiotropy, or whether the associations reflect an early manifestation of neurodevelopmental conditions. Future research could also investigate the role of parental genotypes: whether parental risk alleles for ADHD, autism and schizophrenia are associated with differences in ratings of their children and whether un‐transmitted risk alleles using parent‐child trios contribute to “genetic nurture” effects (Kong et al., 2018). Finally, our sample included only participants of European ancestry and our findings may not generalise to other groups: we encourage future work using samples of different ancestries.

In conclusion, this study examined whether toddler motor development, language development and temperament, previously identified as early indicators of neurodevelopmental conditions, were associated with genetic liability to ADHD, autism and schizophrenia in the general population, as indexed by polygenic scores. We found evidence of association with higher gross motor scores, activity and approach in toddlerhood for ADHD PGS and with greater negative affect for schizophrenia PGS. We did not find strong evidence for associations with autism PGS. Our findings suggest that specific aspects of temperament at age 2 years could represent early manifestations of genetic risk for neurodevelopmental conditions.

## AUTHOR CONTRIBUTIONS

**Lucy Riglin**: Formal analysis, Methodology, Writing – original draft. **Esther Tobarra‐Sanchez**: Conceptualization, Funding acquisition, Writing – original draft. **Evie Stergiakouli**: Writing – review & editing. **Alexandra Havdahl**: Writing – review & editing. **Kate Tilling**: Writing – review & editing. **Michael O’Donovan**: Writing – review & editing. **Joel Nigg**: Writing – review & editing. **Kate Langley**: Conceptualization, Funding acquisition, Supervision, Writing – review & editing. **Anita Thapar**: Conceptualization, Funding acquisition, Writing – original draft.

## CONFLICT OF INTEREST

Kate Tilling has acted as a consultant for CHDI foundation. Joel Nigg and Kate Langley both serve on the JCPP *Advances* Editorial Advisory Board. The remaining authors have declared that they have no competing or potential conflicts of interest.

## ETHICS STATEMENT

Ethical approval for the study was obtained from the ALSPAC Ethics and Law Committee and the Local Research Ethics Committees.

## PATIENT CONSENT STATEMENT

Informed consent for the use of data collected via questionnaires and clinics was obtained from participants following the recommendations of the ALSPAC Ethics and Law Committee at the time. Consent for biological samples has been collected in accordance with the Human Tissue Act (2004).

## Supporting information

Supporting Information S1

## Data Availability

ALSPAC data access is through a system of managed open access.
