# Supplementary material for: Early manifestations of genetic liability for ADHD, autism and schizophrenia at ages 18 and 24 months
Source: JCPP Adv. 2022 Jul 21;2(3):e12093. doi: 10.1002/jcv2.12093 (PMC9762693; doi:10.1002/jcv2.12093)
Supplement: Supplementary file 1 — Supporting Information S1 [file JCV2-2-0-s001.docx]

**Supporting Information**

**The Avon Longitudinal Study of Parents and Children (ALSPAC)**

Pregnant women resident in Avon, UK with expected dates of delivery 1st April 1991 to 31st December 1992 were invited to take part in the study. The initial number of pregnancies enrolled is 14,541 (for these at least one questionnaire has been returned or a “Children in Focus” clinic had been attended by 19/07/99). Of these initial pregnancies, there was a total of 14,676 foetuses, resulting in 14,062 live births and 13,988 children who were alive at 1 year of age. When the oldest children were approximately 7 years of age, an attempt was made to bolster the initial sample with eligible cases who had failed to join the study originally. These individuals were not included in our analyses as we focussed on outcomes prior to age 7 years. The sample is relatively homogeneous in regard to ethnicity (approximately 98% White): all participants are from the Bristol area in the United Kingdom.^1^ Please note that the study website contains details of all the data that is available through a fully searchable data dictionary and variable search tool: <http://www.bristol.ac.uk/alspac/researchers/our-data/>. Further details of the study, measures and sample can be found elsewhere.^1-3^

**Generating polygenic scores**

In total 9912 ALSPAC children were genotyped using the Illumina HumanHap500-quad genotyping array. Individuals were excluded on the basis of gender mismatches; minimal or excessive heterozygosity, disproportionate levels of individual missingness (>3%), insufficient sample replication (IBD <0.8), non-European ancestry (assessed by multidimensional scaling analysis and compared with Hapmap II) and cryptic relatedness (IBD > 0.1). SNPs were excluded based on minor allele frequency (<1%), call rate (<95%) or evidence for violations of Hardy-Weinberg equilibrium (P < 5E-7). Imputation was conducted by the ALSPAC team using Impute V2.2.2 against the 1000 genomes reference panel (Phase 1, Version 3: all polymorphic SNPs excluding singletons), using all 2186 reference haplotypes (including non-Europeans). SNPs were subsequently filtered based on minor allele frequency (<1%) and imputation quality (INFO<0.8). Sensitivity analyses utilised the top 10 principal components (in-line with previously work^4^) generated using PLINK^5^ --pca command (https://gist.github.com/explodecomputer/ab3552ee06b5c0df76139fc587373599). Following quality control and limiting individuals to one child per family, genetic data were available for N=7975.

Genome-wide association study (GWAS) were filtered to remove SNPs that were palindromic, insertions/deletions, non-autosomal, INFO score <0.8, missing in N>1 study and duplicates (<https://github.com/ricanney>).

PGS were generated for individuals in ALSPAC as the number of disorder risk alleles – defined using the GWAS summary statistics - weighted by effect size, using PRSice;^6^ SNPs were clumped with an R^2^ threshold of 0.1 and a distance threshold of 1000kb and excluding the extended major histocompatibility complex (MHC; chromosome 6: 26-33Mb) due to the high linkage disequilibrium (LD) within this region. PGS were derived based on a seven p-value thresholds (p<1, p<0.5, p<0.01, p<0.05, p<0.01, p<0.005, p<0.001), standardized using Z-score transformation and the first principal component extracted and analysed using the PGS–PCA approach.^7^

**Inverse probability weighting**

Inverse probability weighting (IPW) was used to assess the impact of missing genetic data and has been recommended over alternative methods such as multiple imputation in situations where whole blocks of data are missing for a large proportion of individuals.^8^ Weights were derived from a logistic regression analysis of missing genetic data for a set of measures assessed in pregnancy with minimal (<12%) missingness: see Supplementary Table 11. Missing data on indicators used to derive weights were singly imputed as the modal or mean value. The Hosmer-Lemeshow test did not indicate poor fit (Hosmer-Lemeshow χ2(10)=12.83, p=0.12). For the included sample (N=7498) weights ranged from 1.33 to 4.75. Analyses conducted using IPW to minimise potential bias caused by only a subsample of having genetic data revealed the same pattern of results as the primary analyses (see Supplementary Table 7).

**Excluding participants who developed ADHD**

Sensitivity analyses were conducting excluding individuals who met DSM-IV criteria for ADHD.

ADHD was assessed at age 7 years using the Development and Wellbeing Assessment (DAWBA).^9^ A paper version of the DAWBA structured assessment was completed by parents and teachers. Those at high risk were identified by an algorithm and reviewed by child psychiatrists. Coexisting pervasive developmental disorders and affective disorders were excluded to determine a final DSM-IV diagnosis of ADHD. These DAWBA data were available for N=5503 of our sample, of whom N=115 we assigned a diagnosis of ADHD (2%) and N=18 a diagnosis of pervasive developmental disorder: the remaining N=5370 who were assigned as not meeting ADHD diagnostic criteria were included in our sensitivity analyses.

Results (shown in Supplementary Table 9) showed a similar pattern of results to the primary analyses.

| **Table S1.** Multivariable associations between polygenic risk scores and toddler developmental outcomes | | | | | | | | | | | |
| --- | --- | --- | --- | --- | --- | --- | --- | --- | --- | --- | --- |
|  |  | ADHD PGS | | | Autism PGS | | | Schizophrenia PGS | | | Overall model |
|  | N | β | (95% CI) | p | β | (95% CI) | p | β | (95% CI) | p | R^2^ |
| *Motor* |  |  |  |  |  |  |  |  |  |  |  |
| Fine motor | 6301 | -0.01 | (-0.04, 0.01) | 0.28 | 0.01 | (-0.01, 0.04) | 0.27 | -0.02 | (-0.05, 0.01) | 0.08 | 0.08% |
| Gross motor | 6328 | **0.04** | **(0.01, 0.06)** | **3x10^-03^** | -0.02 | (-0.05, 0.00) | 0.09 | -0.02 | (-0.05, 0.00) | 0.11 | 0.19% |
| *Language* |  |  |  |  |  |  |  |  |  |  |  |
| Vocabulary | 6289 | -0.03 | (-0.05, -0.00) | 0.05 | -0.01 | (-0.03, 0.02) | 0.68 | 0.01 | (-0.02, 0.03) | 0.46 | 0.08% |
| Grammar | 5880 | -0.01 | (-0.04, 0.01) | 0.28 | -0.02 | (-0.05, 0.01) | 0.16 | 0.02 | (-0.01, 0.05) | 0.13 | 0.11% |
| *Temperament* |  |  |  |  |  |  |  |  |  |  |  |
| Activity | 6260 | **0.07** | **(0.04, 0.09)** | **5x10^-07^** | -0.01 | -0.03, 0.02) | 0.59 | 0.00 | (-0.02, 0.03) | 0.94 | 0.41% |
| Rhythmicity | 6257 | 0.03 | (0.00, 0.05) | 0.03 | -0.02 | (-0.04, 0.01) | 0.19 | -0.02 | (-0.03, 0.02) | 0.89 | 0.09% |
| Withdrawal | 6253 | **-0.05** | **(-0.07, -0.02)** | **3x10^-04^** | -0.01 | (-0.03, 0.02) | 0.67 | -0.00 | (-0.03, 0.02) | 0.98 | 0.24% |
| Adaptability | 6233 | 0.02 | (-0.01, 0.04) | 0.16 | 0.00 | (-0.03, 0.03) | 0.97 | 0.02 | (-0.00, 0.05) | 0.09 | 0.08% |
| Intensity | 6255 | 0.03 | (0.03, 0.01) | 0.02 | 0.02 | (-0.01, 0.04) | 0.23 | 0.02 | (-0.00, 0.05) | 0.09 | 0.20% |
| Mood | 6259 | -0.01 | (-0.03, 0.02) | 0.57 | -0.01 | (-0.04, 0.02) | 0.42 | **0.04** | **(0.02, 0.07)** | **5x10^-04^** | 0.21% |
| Persistence | 6252 | 0.01 | (-0.02, 0.03) | 0.55 | -0.02 | (-0.05, 0.00) | 0.11 | 0.03 | (0.00, 0.05) | 0.03 | 0.11% |
| Distractibility | 6255 | -0.00 | (-0.03, 0.03) | >0.99 | -0.02 | (-0.05, 0.00) | 0.07 | 0.02 | (-0.00, 0.04) | 0.12 | 0.09% |
| Threshold of response | 6260 | 0.01 | (-0.02, 0.03) | 0.59 | -0.03 | (-0.06, -0.00) | 0.02 | 0.02 | (-0.00, 0.05) | 0.07 | 0.14% |
| Standardised betas presented: effect size reflect 1 SD increase in the outcome variable per 1 SD increase in polygenic risk score. Highlighted in bold where associations meet the multiple testing corrected p-value threshold of <0.005. | | | | | | | | | | | |

| **Table S2.** Principal component analysis of outcomes to identify the number of independent tests | | | | |
| --- | --- | --- | --- | --- |
| Component | Eigenvalue | Difference | Proportion | Cumulative |
| 1 | 2.82 | 0.83 | 0.22 | 0.22 |
| 2 | 1.98 | 0.70 | 0.15 | 0.37 |
| 3 | 1.29 | 0.11 | 0.10 | 0.47 |
| 4 | 1.17 | 0.13 | 0.09 | 0.56 |
| 5 | 1.04 | 0.14 | 0.08 | 0.64 |
| 6 | 0.90 | 0.04 | 0.07 | 0.71 |
| 7 | 0.86 | 0.11 | 0.07 | 0.77 |
| 8 | 0.74 | 0.10 | 0.06 | 0.83 |
| 9 | 0.65 | 0.10 | 0.05 | 0.88 |
| 10 | 0.55 | 0.09 | 0.04 | 0.92 |
| 11 | 0.46 | 0.11 | 0.04 | 0.96 |
| 12 | 0.35 | 0.16 | 0.03 | 0.99 |
| 13 | 0.19 | . | 0.01 | 1.00 |
| N=5411, unrotated rho=1 | | | | |

| **Table S3.** Correlations between toddler developmental outcomes | | | | | | | | | | | | | |
| --- | --- | --- | --- | --- | --- | --- | --- | --- | --- | --- | --- | --- | --- |
|  | A | B | C | D | E | F | G | H | I | J | K | L | M |
| 1. Fine motor | 1 |  |  |  |  |  |  |  |  |  |  |  |  |
| 1. Gross motor | 0.29 | 1 |  |  |  |  |  |  |  |  |  |  |  |
| 1. Vocabulary | 0.27 | 0.20 | 1 |  |  |  |  |  |  |  |  |  |  |
| 1. Grammar | 0.20 | 0.16 | 0.81 | 1 |  |  |  |  |  |  |  |  |  |
| 1. Activity | -0.03 | 0.11 | -0.06 | -0.06 | 1 |  |  |  |  |  |  |  |  |
| 1. Rhythmicity | -0.02 | 0.01 | -0.05 | -0.02 | 0.11 | 1 |  |  |  |  |  |  |  |
| 1. Withdrawal | -0.02 | -0.08 | -0.05 | -0.04 | -0.10 | 0.03 | 1 |  |  |  |  |  |  |
| 1. Adaptability | -0.11 | -0.05 | -0.16 | -0.15 | 0.47 | 0.22 | 0.20 | 1 |  |  |  |  |  |
| 1. Intensity | -0.00 | 0.03 | -0.03 | -0.04 | 0.41 | 0.14 | 0.02 | 0.39 | 1 |  |  |  |  |
| 1. Mood | -0.08 | -0.07 | -0.12 | -0.09 | 0.26 | 0.26 | 0.37 | 0.59 | 0.36 | 1 |  |  |  |
| 1. Persistence | -0.20 | -0.11 | -0.24 | -0.24 | 0.28 | 0.14 | 0.06 | 0.33 | 0.13 | 0.29 | 1 |  |  |
| 1. Distractibility | -0.02 | -0.01 | 0.00 | -0.01 | 0.15 | -0.00 | -0.01 | 0.05 | 0.13 | 0.06 | 0.14 | 1 |  |
| 1. Threshold of response | 0.11 | 0.09 | 0.21 | 0.17 | -0.01 | -0.03 | -0.11 | -0.04 | 0.12 | 0.02 | -0.11 | 0.20 | 1 |
| N=5880-6328 | | | | | | | | | | | | | |

| **Table S4.** Multivariable associations between polygenic scores and toddler developmental outcomes stratified by sex | | | | | | | | | | | |
| --- | --- | --- | --- | --- | --- | --- | --- | --- | --- | --- | --- |
|  |  | ADHD PGS | | | Autism PGS | | | Schizophrenia PGS | | | Overall model |
|  | N | β | (95% CI) | p | β | (95% CI) | p | β | (95% CI) | p | R^2^ |
| a) Males |  |  |  |  |  |  |  |  |  |  |  |
| Fine motor | 3241 | -0.04 | (-0.07, -0.00) | 0.04 | 0.03 | (-0.01, 0.06) | 0.11 | -0.02 | (-0.05, 0.02) | 0.27 | 0.22% |
| Gross motor | 3250 | **0.05** | **(0.02, 0.09)** | **3x10^-03^** | 0.00 | (-0.03, 0.04) | 0.97 | -0.04 | (-0.07, -0.00) | 0.03 | 0.40% |
| Vocabulary | 3232 | -0.00 | (-0.05, 0.03) | 0.59 | -0.02 | (-0.05, 0.02) | 0.33 | -0.00 | (-0.04, 0.03) | 0.91 | 0.05% |
| Grammar | 2951 | -0.01 | (-0.04, 0.03) | 0.72 | -0.03 | (-0.07, 0.00) | 0.07 | 0.01 | (-0.03, 0.05) | 0.63 | 0.14% |
| Activity | 3222 | **0.07** | **(0.04, 0.11)** | **8x10^-05^** | -0.02 | (-0.06, 0.01) | 0.25 | -0.00 | (-0.04, 0.03) | 0.25 | 0.48% |
| Rhythmicity | 3221 | 0.04 | (0.01, 0.08) | 0.02 | -0.01 | (-0.04, 0.04) | 0.74 | -0.01 | (0.04, 0.03) | 0.75 | 0.17% |
| Withdrawal | 3219 | **-0.06** | **(-0.10, -0.02)** | **1x10^-03^** | 0.00 | (-0.03, 0.04) | 0.83 | 0.00 | (-0.03, 0.04) | 0.95 | 0.35% |
| Adaptability | 3209 | 0.02 | (-0.01, 0.06) | 0.21 | -0.00 | (-0.04, 0.03) | 0.81 | 0.01 | (-0.02, 0.05) | 0.43 | 0.07% |
| Intensity | 3218 | 0.02 | (-0.01, 0.06) | 0.23 | 0.00 | (-0.03, 0.04) | 0.80 | 0.02 | (-0.01, 0.06) | 0.23 | 0.11% |
| Mood | 3221 | -0.01 | (-0.04, 0.03) | 0.71 | -0.02 | (-0.05, 0.02) | 0.31 | 0.04 | (0.01, 0.07) | 0.02 | 0.19% |
| Persistence | 3219 | 0.02 | (-0.01, 0.06) | 0.19 | -0.03 | (-0.06, 0.01) | 0.15 | 0.04 | (0.00, 0.07) | 0.03 | 0.25% |
| Distractibility | 3219 | -0.01 | (-0.05, 0.02) | 0.43 | -0.04 | (-0.07, -0.00) | 0.04 | 0.03 | (-0.01, 0.06) | 0.12 | 0.25% |
| Threshold of response | 3221 | -0.01 | (-0.04, 0.03) | 0.64 | -0.01 | (-0.05, 0.02) | 0.43 | 0.03 | (-0.00, 0.07) | 0.06 | 0.14% |
| b) Females |  |  |  |  |  |  |  |  |  |  |  |
| Fine motor | 3060 | 0.01 | (-0.02, 0.05) | 0.53 | -0.00 | (-0.04, 0.04) | 0.95 | -0.02 | (-0.06, 0.01) | 0.18 | 0.07% |
| Gross motor | 3078 | 0.02 | (-0.01, 0.06) | 0.21 | -0.04 | (-0.08, -0.01) | 0.02 | -0.00 | (-0.04, 0.03) | 0.87 | 0.20% |
| Vocabulary | 3057 | -0.05 | (-0.08, -0.00) | 0.03 | 0.00 | (-0.03, 0.04) | 0.85 | 0.03 | (-0.01, 0.06) | 0.15 | 0.22% |
| Grammar | 2929 | -0.02 | (-0.06, 0.02) | 0.32 | -0.01 | (-0.05, 0.03) | 0.66 | 0.03 | (-0.00, 0.07) | 0.08 | 0.15% |
| Activity | 3038 | **0.06** | **(0.02, 0.09)** | **2x10^-03^** | 0.01 | (-0.03, 0.05) | 0.64 | 0.00 | (-0.03, 0.04) | 0.85 | 0.38% |
| Rhythmicity | 3036 | 0.01 | (-0.02, 0.05) | 0.45 | -0.03 | (-0.07, 0.01) | 0.12 | 0.00 | (-0.03, 0.04) | 0.12 | 0.09% |
| Withdrawal | 3034 | -0.03 | (-0.07, 0.00) | 0.08 | -0.02 | (-0.05, 0.02) | 0.36 | 0.00 | (-0.04, 0.04) | 0.99 | 0.16% |
| Adaptability | 3024 | 0.01 | (-0.02, 0.05) | 0.50 | 0.01 | (-0.03, 0.04) | 0.71 | 0.03 | (-0.01, 0.06) | 0.13 | 0.11% |
| Intensity | 3037 | 0.04 | (0.00, 0.08) | 0.03 | 0.03 | (-0.01, 0.06) | 0.15 | 0.02 | (-0.01, 0.06) | 0.24 | 0.35% |
| Mood | 3038 | -0.01 | (-0.04, 0.03) | 0.67 | -0.02 | (-0.04, 0.03) | 0.92 | **0.05** | **(0.01, 0.08)** | **8x10^-03^** | 0.24% |
| Persistence | 3033 | -0.01 | (-0.05, 0.03) | 0.57 | -0.01 | (-0.05, 0.02) | 0.44 | 0.01 | (-0.02, 0.05) | 0.48 | 0.05% |
| Distractibility | 3036 | 0.02 | (-0.02, 0.05) | 0.39 | -0.01 | (-0.05, 0.03) | 0.61 | 0.01 | (-0.02, 0.05) | 0.50 | 0.04% |
| Threshold of response | 3039 | 0.03 | (-0.01, 0.06) | 0.18 | -0.05 | (-0.09, -0.01) | 0.01 | 0.02 | (-0.02, 0.05) | 0.39 | 0.27% |
| Standardised betas presented: effect size reflect 1 SD increase in the outcome variable per 1 SD increase in polygenic risk score. Highlighted in bold where associations meet the multiple testing corrected p-value threshold of <0.005. | | | | | | | | | | | |

| **Table S5.** Interaction terms assessing sex-differences in multivariable associations between polygenic risk scores and toddler developmental | | | | | | | | | | |
| --- | --- | --- | --- | --- | --- | --- | --- | --- | --- | --- |
|  |  | ADHD PGS | | | Autism PGS | | | Schizophrenia PGS | | |
|  | N | β | (95% CI) | p | β | (95% CI) | p | β | (95% CI) | p |
| *Motor* |  |  |  |  |  |  |  |  |  |  |
| Fine motor | 6301 | -0.05 | (-0.10, 0.00) | 0.05 | 0.03 | (-0.02, 0.08) | 0.23 | 0.00 | (-0.05, 0.05) | 0.87 |
| Gross motor | 6328 | 0.03 | (-0.02, 0.08) | 0.27 | 0.05 | (-0.01, 0.10) | 0.08 | -0.03 | (-0.08, 0.02) | 0.18 |
| *Language* |  |  |  |  |  |  |  |  |  |  |
| Vocabulary | 6289 | 0.03 | (-0.02, 0.08) | 0.25 | -0.02 | (-0.07, 0.03) | 0.40 | -0.03 | (-0.08, 0.02) | 0.28 |
| Grammar | 5880 | 0.01 | (-0.04, 0.07) | 0.63 | -0.03 | (-0.08, 0.03) | 0.34 | -0.02 | (-0.07, 0.03) | 0.34 |
| *Temperament* |  |  |  |  |  |  |  |  |  |  |
| Activity | 6260 | 0.01 | (-0.04, 0.06) | 0.63 | -0.03 | (-0.08, 0.02) | 0.25 | -0.01 | (-0.06, 0.04) | 0.79 |
| Rhythmicity | 6257 | 0.03 | (-0.02, 0.08) | 0.27 | 0.02 | (-0.03, 0.07) | 0.40 | -0.01 | (-0.06, 0.04) | 0.74 |
| Withdrawal | 6253 | -0.03 | (-0.08, 0.02) | 0.29 | 0.02 | (-0.03, 0.07) | 0.43 | 0.00 | (-0.05, 0.05) | 0.98 |
| Adaptability | 6233 | 0.01 | (-0.04, 0.06) | 0.70 | -0.01 | (-0.06, 0.04) | 0.66 | -0.01 | (-0.06, 0.04) | 0.58 |
| Intensity | 6255 | -0.02 | (-0.07, 0.03) | 0.49 | -0.02 | (-0.07, 0.03) | 0.41 | 0.00 | (-0.05, 0.05) | 0.98 |
| Mood | 6259 | 0.00 | (-0.05, 0.05) | 0.98 | -0.02 | (-0.07, 0.03) | 0.51 | -0.01 | (-0.06, 0.04) | 0.78 |
| Persistence | 6252 | 0.03 | (-0.02, 0.09) | 0.19 | -0.01 | (-0.06, 0.04) | 0.63 | 0.03 | (-0.02, 0.08) | 0.31 |
| Distractibility | 6255 | -0.03 | (-0.08, 0.02) | 0.24 | -0.03 | (-0.08, 0.02) | 0.25 | 0.02 | (-0.03, 0.07) | 0.52 |
| Threshold of response | 6260 | -0.03 | (-0.08, 0.02) | 0.20 | 0.03 | (-0.02, 0.09) | 0.18 | 0.02 | (-0.03, 0.07) | 0.49 |
| Standardised betas presented: effect size reflect 1 SD increase in the outcome variable per 1 SD increase in polygenic risk score | | | | | | | | | | |

| **Table S6.** Univariable associations between polygenic scores and toddler developmental | | | | | | | | | | |
| --- | --- | --- | --- | --- | --- | --- | --- | --- | --- | --- |
|  |  | ADHD PGS | | | Autism PGS | | | Schizophrenia PGS | | |
|  | N | β | (95% CI) | p | β | (95% CI) | p | β | (95% CI) | p |
| *Motor* |  |  |  |  |  |  |  |  |  |  |
| Fine motor | 6301 | -0.01 | (-0.04, 0.01) | 0.34 | 0.01 | (-0.01, 0.03) | 0.42 | -0.02 | (-0.05, 0.00) | 0.07 |
| Gross motor | 6328 | 0.03 | (0.01, 0.06) | 0.01 | -0.01 | (-0.04, 0.01) | 0.28 | -0.02 | (-0.04, 0.01) | 0.13 |
| *Language* |  |  |  |  |  |  |  |  |  |  |
| Vocabulary | 6289 | -0.03 | (-0.05 -0.00) | 0.04 | -0.01 | (-0.04, 0.01) | 0.37 | 0.01 | (-0.02, 0.03) | 0.54 |
| Grammar | 5880 | -0.02 | (-0.04, 0.01) | 0.16 | -0.02 | (-0.05, 0.00) | 0.09 | 0.02 | (-0.01, 0.04) | 0.16 |
| *Temperament* |  |  |  |  |  |  |  |  |  |  |
| Activity | 6260 | 0.06 | (0.04, 0.09) | 4x10^-07^ | 0.01 | (-0.02, 0.03) | 0.47 | 0.00 | (-0.02, 0.03) | 0.74 |
| Rhythmicity | 6257 | 0.02 | (-0.00, 0.05) | 0.05 | -0.01 | (-0.03, 0.01) | 0.43 | -0.00 | (-0.03, 0.02) | 0.95 |
| Withdrawal | 6253 | -0.05 | (-0.07, -0.02) | 1x10^-04^ | -0.02 | (-0.04, 0.01) | 0.17 | 0.00 | (-0.03, 0.02) | 0.82 |
| Adaptability | 6233 | 0.02 | (-0.01, 0.04) | 0.12 | 0.01 | (-0.02, 0.03) | 0.65 | 0.02 | (-0.00, 0.05) | 0.08 |
| Intensity | 6255 | 0.04 | (-0.01, 0.06) | 4x10^-03^ | 0.02 | (-0.00, 0.05) | 0.06 | 0.02 | (-0.00, 0.05) | 0.06 |
| Mood | 6259 | -0.01 | (-0.03, 0.02) | 0.55 | -0.01 | (-0.04, 0.01) | 0.39 | 0.04 | (-0.02, 0.07) | 1x10^-03^ |
| Persistence | 6252 | 0.00 | (-0.02, 0.03) | 0.76 | -0.02 | (-0.04, 0.01) | 0.16 | 0.03 | (-0.00, 0.05) | 0.04 |
| Distractibility | 6255 | 0.00 | (-0.03, 0.02) | 0.67 | -0.02 | (-0.05, 0.00) | 0.07 | 0.02 | (-0.01, 0.04) | 0.13 |
| Threshold of response | 6260 | 0.01 | (-0.02, 0.03) | 0.96 | -0.03 | (-0.05, -0.00) | 0.03 | 0.02 | (-0.00, 0.05) | 0.08 |
| Standardised betas presented: effect size reflect 1 SD increase in the outcome variable per 1 SD increase in polygenic risk score | | | | | | | | | | |

| **Table S7.** Multivariable associations using inverse probability weighting | | | | | | | | | | |
| --- | --- | --- | --- | --- | --- | --- | --- | --- | --- | --- |
|  |  | ADHD PGS | | | Autism PGS | | | Schizophrenia PGS | | |
|  | N | β | (95% CI) | p | β | (95% CI) | p | β | (95% CI) | p |
| *Motor* |  |  |  |  |  |  |  |  |  |  |
| Fine motor | 6301 | -0.02 | (-0.04, 0.01) | 0.23 | 0.01 | (-0.01, 0.04) | 0.34 | -0.02 | (-0.05, 0.00) | 0.12 |
| Gross motor | 6328 | 0.04 | (0.01, 0.06) | 7x10^-03^ | -0.02 | (-0.05, 0.00) | 0.09 | -0.01 | (-0.04, 0.01) | 0.24 |
| *Language* |  |  |  |  |  |  |  |  |  |  |
| Vocabulary | 6289 | -0.03 | (-0.05, -0.00) | 0.04 | -0.00 | (-0.03, 0.02) | 0.77 | 0.01 | (-0.01, 0.04) | 0.32 |
| Grammar | 5880 | -0.01 | (-0.04, 0.01) | 0.32 | -0.02 | (-0.05, 0.01) | 0.15 | 0.02 | (-0.01, 0.05) | 0.13 |
| *Temperament* |  |  |  |  |  |  |  |  |  |  |
| Activity | 6260 | 0.06 | (0.04, 0.09) | 8x10^-07^ | -0.00 | (-0.03, 0.02) | 0.72 | -0.00 | (-0.03, 0.02) | 0.96 |
| Rhythmicity | 6257 | 0.03 | (0.00, 0.05) | 0.03 | -0.02 | (-0.04, 0.01) | 0.15 | -0.00 | (-0.03, 0.02) | 0.78 |
| Withdrawal | 6253 | -0.05 | (-0.07, -0.02) | 3x10^-04^ | -0.01 | (-0.03, 0.02) | 0.66 | 0.00 | (-0.02, 0.03) | 0.81 |
| Adaptability | 6233 | 0.02 | (-0.01, 0.05) | 0.14 | -0.00 | (-0.03, 0.02) | 0.89 | 0.02 | (-0.00, 0.05) | 0.09 |
| Intensity | 6255 | 0.03 | (0.00, 0.06) | 0.02 | 0.01 | (-0.01, 0.04) | 0.36 | 0.02 | (-0.00, 0.05) | 0.09 |
| Mood | 6259 | -0.01 | (-0.03, 0.02) | 0.52 | -0.01 | (-0.04, 0.02) | 0.44 | 0.04 | (0.02, 0.07) | 1x10^-03^ |
| Persistence | 6252 | 0.01 | (-0.02, 0.04) | 0.45 | -0.02 | (-0.04, 0.01) | 0.15 | 0.03 | (0.00, 0.05) | 0.03 |
| Distractibility | 6255 | -0.00 | (-0.03, 0.02) | 0.88 | -0.02 | (-0.05, 0.00) | 0.10 | 0.02 | (-0.00, 0.05) | 0.09 |
| Threshold of response | 6260 | 0.00 | (-0.03, 0.03) | 0.95 | -0.03 | (-0.06, -0.01) | 0.02 | 0.03 | (0.00, 0.05) | 0.03 |
| Standardised betas presented: effect size reflect 1 SD increase in the outcome variable per 1 SD increase in polygenic risk score | | | | | | | | | | |

| **Table S8.** Multivariable associations between polygenic scores and toddler developmental including population stratification covariates | | | | | | | | | | |
| --- | --- | --- | --- | --- | --- | --- | --- | --- | --- | --- |
|  |  | ADHD PGS | | | Autism PGS | | | Schizophrenia PGS | | |
|  | N | β | (95% CI) | p | β | (95% CI) | p | β | (95% CI) | p |
| *Motor* |  |  |  |  |  |  |  |  |  |  |
| Fine motor | 6297 | -0.01 | (-0.04, 0.01) | 0.28 | 0.02 | (-0.01, 0.04) | 0.24 | -0.02 | (-0.05, 0.01) | 0.08 |
| Gross motor | 6324 | **0.04** | **(0.01, 0.06)** | **3x10^-03^** | -0.02 | (-0.05, 0.00) | 0.10 | -0.02 | (-0.04, 0.01) | 0.15 |
| *Language* |  |  |  |  |  |  |  |  |  |  |
| Vocabulary | 6285 | -0.03 | (-0.05, -0.00) | 0.05 | -0.00 | (-0.03, 0.02) | 0.71 | 0.01 | (-0.02, 0.03) | 0.52 |
| Grammar | 5878 | -0.01 | (-0.04, 0.01) | 0.30 | -0.02 | (-0.05, 0.01) | 0.15 | 0.02 | (-0.01, 0.04) | 0.17 |
| *Temperament* |  |  |  |  |  |  |  |  |  |  |
| Activity | 6256 | **0.07** | **(0.04, 0.09)** | **4x10^-07^** | -0.01 | -0.03, 0.02) | 0.56 | 0.00 | (-0.02, 0.03) | 0.98 |
| Rhythmicity | 6253 | 0.03 | (0.00, 0.05) | 0.03 | -0.02 | (-0.04, 0.01) | 0.18 | -0.00 | (-0.03, 0.02) | 0.90 |
| Withdrawal | 6249 | **-0.05** | **(-0.07, -0.02)** | **4x10^-04^** | -0.00 | (-0.03, 0.02) | 0.73 | -0.00 | (-0.03, 0.02) | 0.97 |
| Adaptability | 6229 | 0.02 | (-0.01, 0.04) | 0.15 | -0.00 | (-0.03, 0.03) | 0.97 | 0.02 | (-0.00, 0.05) | 0.10 |
| Intensity | 6251 | 0.03 | (0.01, 0.06) | 0.02 | 0.01 | (-0.01, 0.04) | 0.26 | 0.02 | (-0.00, 0.05) | 0.09 |
| Mood | 6255 | -0.01 | (-0.03, 0.02) | 0.57 | -0.01 | (-0.04, 0.02) | 0.42 | **0.04** | **(0.02, 0.07)** | **5x10^-04^** |
| Persistence | 6248 | 0.01 | (-0.02, 0.03) | 0.51 | -0.02 | (-0.05, 0.00) | 0.10 | 0.03 | (0.00, 0.05) | 0.04 |
| Distractibility | 6251 | -0.00 | (-0.03, 0.02) | 0.96 | -0.02 | (-0.05, 0.00) | 0.07 | 0.02 | (-0.00, 0.05) | 0.11 |
| Threshold of response | 6256 | 0.01 | (-0.02, 0.03) | 0.58 | -0.03 | (-0.06, -0.00) | 0.02 | 0.02 | (-0.00, 0.05) | 0.06 |
| Standardised betas presented: effect size reflect 1 SD increase in the outcome variable per 1 SD increase in polygenic risk score. Highlighted in bold where associations meet the multiple testing corrected p-value threshold of <0.005. | | | | | | | | | | |

| **Table S9.** Multivariable associations excluding those with ADHD | | | | | | | | | | |
| --- | --- | --- | --- | --- | --- | --- | --- | --- | --- | --- |
|  |  | ADHD PGS | | | Autism PGS | | | Schizophrenia PGS | | |
|  | N | β | (95% CI) | p | β | (95% CI) | p | β | (95% CI) | p |
| *Motor* |  |  |  |  |  |  |  |  |  |  |
| Fine motor | 4987 | -0.01 | (-0.03, 0.02) | 0.72 | 0.01 | (-0.02, 0.04) | 0.65 | -0.03 | (-0.05, 0.00) | 0.06 |
| Gross motor | 5005 | 0.04 | (0.01, 0.07) | 9x10^-03^ | -0.02 | (-0.05, 0.01) | 0.18 | -0.02 | (-0.05, 0.00) | 0.10 |
| *Language* |  |  |  |  |  |  |  |  |  |  |
| Vocabulary | 5057 | -0.02 | (-0.05, 0.01) | 0.13 | -0.00 | (-0.03, 0.03) | 0.98 | 0.02 | (-0.01, 0.05) | 0.18 |
| Grammar | 4748 | -0.01 | (-0.04, 0.02) | 0.70 | -0.01 | (-0.04, 0.02) | 0.35 | 0.02 | (-0.01, 0.05) | 0.22 |
| *Temperament* |  |  |  |  |  |  |  |  |  |  |
| Activity | 5040 | 0.07 | (0.05, 0.10) | 4x10^-07^ | -0.01 | (-0.04, 0.02) | 0.51 | 0.01 | (-0.02, 0.03) | 0.60 |
| Rhythmicity | 5040 | 0.03 | (0.00, 0.06) | 0.02 | -0.01 | (-0.03, 0.02) | 0.67 | -0.01 | (-0.04, 0.02) | 0.51 |
| Withdrawal | 5039 | -0.05 | (-0.08, -0.02) | 5x10^-04^ | -0.01 | (-0.03, 0.02) | 0.68 | -0.00 | (-0.03, 0.03) | 0.97 |
| Adaptability | 6233 | 0.03 | (0.00, 0.06) | 0.05 | 0.00 | (-0.02, 0.03) | 0.80 | 0.03 | (-0.02, 0.03) | 0.04 |
| Intensity | 5040 | 0.03 | (0.01, 0.06) | 0.02 | 0.01 | (-0.02, 0.04) | 0.51 | 0.02 | (-0.01, 0.05) | 0.12 |
| Mood | 5040 | 0.00 | (-0.03, 0.03) | 0.93 | -0.01 | (-0.04, 0.02) | 0.62 | 0.05 | (0.02, 0.08) | 2x10^-04^ |
| Persistence | 5037 | 0.01 | (-0.02, 0.03) | 0.66 | -0.02 | (-0.05, 0.01) | 0.13 | 0.04 | (0.01, 0.07) | 5x10^-03^ |
| Distractibility | 5039 | 0.01 | (-0.02, 0.03) | 0.72 | -0.03 | (-0.06, -0.00) | 0.05 | 0.03 | (0.00, 0.06) | 0.05 |
| Threshold of response | 5042 | 0.01 | (-0.02, 0.04) | 0.39 | -0.03 | (-0.06, 0.00) | 0.05 | 0.03 | (0.00, 0.06) | 0.04 |
| Standardised betas presented: effect size reflect 1 SD increase in the outcome variable per 1 SD increase in polygenic risk score | | | | | | | | | | |

| **Table S10.** Questions included in motor assessments | | | |
| --- | --- | --- | --- |
|  | Yes, can  do well | Has only done once  or twice | Has not yet  started |
| *Fine motor questions* |  |  |  |
| 1. Can hold a rattle |  |  |  |
| \| 1. Can focus her eyes on a small object such as a raisin \| \| --- \| |  |  |  |
| 1. Can pick up a small object such as a raisin |  |  |  |
| 1. Can pass an object from one hand to another |  |  |  |
| 1. Can bang together two similar objects that she is holding |  |  |  |
| 1. Grabs objects using the whole hand |  |  |  |
| 1. Can pick up a small object using finger and thumb only |  |  |  |
| 1. Will use a pencil and scribble |  |  |  |
| 1. Can build a tower putting one object on top of another |  |  |  |
| 1. Can build a tower of 3 bricks |  |  |  |
| 1. Can build a tower of 4 bricks |  |  |  |
| 1. Can build a tower 8 bricks |  |  |  |
| 1. Holds a pencil in her fist |  |  |  |
| 1. Can copy a vertical line with a pencil |  |  |  |
| 1. Points to what she wants |  |  |  |
| 1. Will turn the pages of a book |  |  |  |
| *Gross motor questions* |  |  |  |
| 1. Can stand up without being supported even if only for a very short time |  |  |  |
| 1. From a standing position can bend down and return to standing |  |  |  |
| 1. Can stand alone for at least a minute without holding on to anything |  |  |  |
| 1. Can walk while holding someone's hand |  |  |  |
| 1. Can walk alone for at least 5 steps |  |  |  |
| 1. Can walk backwards 5 steps |  |  |  |
| 1. Can kick a ball |  |  |  |
| 1. Can throw a ball |  |  |  |
| 1. Can balance on one foot |  |  |  |
| 1. Can jump up and down |  |  |  |
| 1. Can climb stairs |  |  |  |

| **Table S11.** Associations between variables included in the inverse probability weight and not having genetic data | |
| --- | --- |
|  | Multivariable association |
| Maternal age (years) | OR=0.95, 95% CI=0.95-0.96 |
| Maternal education (1-5) | OR=0.83, 95% CI=0.80-0.85 |
| Crowding index (0-3) | OR=1.09, 95% CI=1.05-1.13 |
| Gestation (weeks) | OR=0.98, 95% CI=0.96-1.00 |
| Birthweight (kgs) | OR=0.80, 95% CI=0.74-0.86 |

**References**

1. Fraser A, Macdonald-Wallis C, Tilling K, Boyd A, Golding J, Davey Smith G *et al.* Cohort Profile: the Avon Longitudinal Study of Parents and Children: ALSPAC mothers cohort. *International journal of epidemiology* 2013; **42**(1)**:** 97-110.

2. Northstone K, Lewcock M, Groom A, Boyd A, Macleod J, Timpson N *et al.* The Avon Longitudinal Study of Parents and Children (ALSPAC): an update on the enrolled sample of index children in 2019. *Wellcome Open Res* 2019; **4:** 51-51.

3. Boyd A, Golding J, Macleod J, Lawlor DA, Fraser A, Henderson J *et al.* Cohort Profile: the 'children of the 90s'--the index offspring of the Avon Longitudinal Study of Parents and Children. *International journal of epidemiology* 2013; **42**(1)**:** 111-127.

4. Martin J, Hamshere ML, Stergiakouli E, O'Donovan MC, Thapar A. Neurocognitive abilities in the general population and composite genetic risk scores for attention-deficit hyperactivity disorder. *J Child Psychol Psychiatry* 2015; **56**(6)**:** 648-656.

5. Chang CC, Chow CC, Tellier LC, Vattikuti S, Purcell SM, Lee JJ. Second-generation PLINK: rising to the challenge of larger and richer datasets. *GigaScience* 2015; **4**(1).

6. Euesden J, Lewis CM, O'Reilly PF. PRSice: Polygenic Risk Score software. *Bioinformatics* 2015; **31**(9)**:** 1466-1468.

7. Coombes BJ, Ploner A, Bergen SE, Biernacka JM. A principal component approach to improve association testing with polygenic risk scores. *Genetic epidemiology* 2020; **44**(7)**:** 676-686.

8. Seaman SR, White IR, Copas AJ, Li L. Combining multiple imputation and inverse-probability weighting. *Biometrics* 2012; **68**(1)**:** 129-137.

9. Goodman R, Ford T, Richards H, Gatward R, Meltzer H. The Development and Well-Being Assessment: description and initial validation of an integrated assessment of child and adolescent psychopathology. *J Child Psychol Psychiatry* 2000; **41**(5)**:** 645-655.
